# Effect of pregnancy on sexual function of couples

**DOI:** 10.4314/ahs.v18i2.5

**Published:** 2018-06

**Authors:** Zahra Bostani Khalesi, Mahshid Bokaie, Seyedeh Maryam Attari

**Affiliations:** 1 Nursing and Midwifery School, Guilan University of Medical Sciences, Rasht, Iran; 2 Research Center for Nursing and Midwifery Care, Shahid Sadoughi University of Medical Sciences, Yazd, Iran; 3 Reproductive Health Research Center, Al-zahra Hospital, Medical School, Guilan University of Medical Sciences, Rasht, Iran

**Keywords:** Sexual activity, pregnant woman, husband

## Abstract

**Objective:**

Sexual function is an important part of each human being's personality and in the general couple relationship, with an obvious impact on quality of life and safe sexual performance during pregnancy is important for couples. The objective of this study was to assess effects of pregnancy on sexual function of couples.

**Materials:**

In a prospective cohort study 123 couples were enrolled in the study when women were first diagnosed to be pregnant. During their pre-natal visits, Sexual function of couples was evaluated using the Iranian Version Index of Erectile Function (IIEF) in men and Female Sexual Function Index (FSFI) in women in three trimesters. Statistical analysis was performed.

**Results:**

Indices of sexual function showed significant regressions over time during pregnancy. The greatness of the problem was highest during the third trimester. Female sexual arousal and sexual satisfaction domain scores had the major correlation to IIEF total score. On the other hand, male intercourse satisfaction domain score had the maximum correlation to FSFI total score. A strong correlation between male and female sexual function was observed.

**Conclusion:**

Sexual function is a widespread problem during pregnancy among Iranian couples. Therefore, pregnant women and their husbands need counseling about healthy sexual function in pregnancy.

## Introduction

Pregnancy is one of the most serious periods in women's lives^1^. Sexuality is an important part of health and well-being^2^. According to the World Health Organization, safe sexual experiences cannot be only defined as the absence of sexual dysfunction, but as a state of physical, emotional, mental, and social well- being related to sexuality^3^. Sexuality is a taboo subject in many countries, including Iran^4^. That negatively affects quality of life and may often be responsible for psychopathological disturbances^5^. Thus, people usually are unwilling to talk about their sexual problems and often, sexual dysfunction goes less than recognized and treated^6^,^7^. Sexual function is an important part of each human being's personality and a keystone in the general couple relationship, with an obvious impact on quality of life^8^.

Pregnancy is a condition which leads to adverse effect on the quality of the sexual relationship between the couples^9^. Safe sexual function during pregnancy is one of the keystones for couples to go forward from partners to parents^10^. In the most literature, it has been shown that there is an association between pregnancy and sexual dysfunction^11^.

Sexual function for any couple has a complex etiology, determined by several different psychological, cultural, ethical, sociological, organic, and neurological factors^12^,^9^. Throughout the gestational period, in addition to the strong influence of hormones, emotional changes may occur in life style, and these may affect the expression of sexual desires and sexual behavior^13^. Both the woman and her partner may worry about complications during pregnancy as a result of sexual activity^14^.

Discomfort of pregnancy can affect the satisfaction of both men and women^15^. Inability to perform up to the partner's expectation in a sexual relationship is usually well thought-out as a faintness^16^.

Marital relationship and couple sexual activity change during pregnancy^17^. The indices of sexual function are affected during pregnancy with a significant decline in the last trimester^18^. The sexual intercourse frequency is low during pregnancy and reaches lower levels in the third trimester in both man and woman^11^. The studies conducted on the issue show that 90% of pregnant women had had no sexual intercourse in the past four weeks. 70% were not concerned about decreasing sexual desire in the pregnancy period^6^. Only 11.2% of pregnant women displayed positive attitudes about sexuality during pregnancy^19^. Based on the results of studies, 68% of pregnant women elicited never having asked their health provider to discuss sexual problems throughout the duration of pregnancy^6^,^7^.

Sexual dysfunction in Iranian pregnant women was rather high. Moreover, men's sexual responses are affected the transition to fatherhood^20^.

There is some evidence that male sexual performance in wife's pregnancy is influenced by both social and psychological factors and decreases towards the end of pregnancy^10^. Several factors may affect and influence the quality of couple's sexual intercourse during pregnancy. Fear of injury to the fetus is the major influence on male sexual activity^7^,^13^. Afraid of hurting a female interfere with desire and arousal in males and therefore can impact on sexual function^9^. Also, females are afraid of insufficient satisfaction of her husband^21^. Despite the increasing number of epidemiologic studies, there is no sufficient data in medical literature regarding prevalence of sexual function among couples during pregnancy. Using this assumption, we used the Iranian Version Index of Erectile Function in men and Female Sexual Function Index in women to comparative evaluation of sexual function in each trimester of pregnancy in couples.

## Materials and methods

Institutional review board approval was obtained before initiation of this study and all participants provided written permission. This prospective cohort study was conducted on a group of healthy women who were first diagnosed to be pregnant and their husbands. All women who came in for their first visit pre-natal care in urban health centers in Rasht from January 2015 to September 2016 were requested to participate, along with their husbands. If the husband was absent at the first prenatal visit and the woman was eligible to participate, they were asked, “Is it possible to come to the Health Center and complete the questionnaire?”

123 couples who had the inclusion criteria were approached.

**Inclusion criteria:** Participants had to be able to read and have a lasting relationship together. To determine the socio-demographic variables such as age, sex, duration of marriage, education, toxic habits, medical history, disability and illness, help seeking, economy, ethnicity, geographic location and pregnancy and labor number and information about their sexual life, a questionnaire was filled. During their prenatal visits, Sexual function of couples was evaluated using the Iranian Version Index of Erectile Function in men^21^ and Female Sexual Function Index^22^ in women. The International Index of Erectile Function (IIEF) is a broadly used, multi-dimensional self-report tool for the assessment of male sexual function. Validity and reliability study of the Iranian Version of IIEF by Pakpour has proven it to be a suitable tool^23^. The instrument is widely accepted by both the regulatory agencies and scientific journals as a valid and reliable measure of sexual functioning in men. It has been linguistically validated and is currently available in 32 languages world-wide. The five domains of the IIEF include erectile function (six items), orgasmic function (two items), sexual desire (two items), intercourse satisfaction and overall satisfaction (two items). Totally items are responded to on a 5-point (for Questions 1 to 10) or 6-point (for Questions 11 to15) Likert-type scale, with higher scores indicating better sexual functioning. Domain scores are derived by adding the point values for each item in the domain together. A simple computational algorithm can be devised to determine the total scale score. In the initial validation study, the IIEF was shown to have a high degree of internal reliability (Cronbach's α >. 82) and high test-retest reliability for each domain. The Female Sexual Function Index is a validated and reliable measure for female sexual function. This standardized has 19 questions and assesses six dominions of female sexual functioning, including desire, arousal, lubrication, orgasm, satisfaction and pain during the last 4 weeks. The FSFI is a valid and accurate measure of the female sexual function. This questionnaire comprises 19 questions that evaluate six different domains of sexual function, including desire, arousal, lubrication, orgasm, satisfaction and pain. Questions 1, 2, 15 and 16 were scored from 1 to 5; all the others were scored from 0 to 5. Each domain score was obtained by adding individual items of the domain and multiplying this result by the domain factor (i.e. Desire, 0.6; arousal and lubrication, 0.3; orgasm, satisfaction and pain, 0.4). The FSFI total score is determined by the sum of the six domains and can vary from 2 to 36, where higher scores are associated with the lower degree of sexual dysfunction. Since a total score of 26 is the cutoff point for women with sexual dysfunction, the present study considered patients that were scored 26 and under as presenting the disorder^24^. The FSFI information was translated to Persian and previously validated for Iranian women. Validity and reliability study of the Iranian Version of FSFI by Fakhri has proven it to be a suitable. The overall test-retest reliability coefficients were high for each domain of the Iranian Version of FSFI (0.73 to 0.86) and the internal consistencies within the acceptable range (Cronbach's α >. 90)^25^.

All male participants were asked to complete Index of Erectile Function and female participants FSFI form during the pre-natal visits, in each trimester. Pre-natal care was performed throughout the pregnancy. The pregnant women with fetal-maternal complication or associated conditions during pregnancy that required abstinence from sexual activity were excluded. Gestational age was determined from the last menstrual cycle and was verified by ultrasound scan measurements.

All data were analyzed using SPSS software version 19.0 (SPSS Inc., Chicago, IL, USA). The data were existing as mean ± SD or percentages. The relationship between male and female sexual function using correlation analysis was performed. Categorical and continuous data were shown by the number (percentage), mean, standard deviation and median [min, max], respectively. The first trimester and the second trimester were compared using the Mann-Whitney test. The second trimester and the third trimester using the Friedman test and the Wilcoxon test (between two consecutive trimesters).The difference at P <0.05 was considered significant.

## Results

The initial sample had 178 couples approached, however 55 were excluded as described : 21 patients in the first trimester (6 miscarriages, 13 miscarriage threats, 2 hospital admission due to hyperemesis gravidarum), 9 in the second (2 miscarriages, 2 miscarriage threats, 1 Preeclampsia, 4 Failure to participate) and 25 in the third trimester (11 premature labors, 4 premature ruptures of membranes, 3 genital bleedings, 2 hospital admission due to severe preeclampsia, 5 Failure to participate) were excluded because they presented with clinical conditions restricting sexual activity during pregnancy. Couples as well as the mean duration of the marriage were 26.8 ± 5.2 years and 5.2 ± 1.6 years respectively.

The husbands (31.1 ± 8.6) were significantly older (p < 0.0001) than their corresponding wives (27.3 ± 2.2). The findings showed that there was no significant association between sexual dysfunction and age. Whereas 22.8% of the husbands had attained higher education and 67.2% was nulliparous. The FSFI average scores were compared between the gestational trimesters using for the first comparison the Mann-Whitney non-parametric test; for the second comparison, the Friedman test (in the three trimesters) and the Wilcoxon test (between two consecutive trimesters). Then, the average scores were compared in the six FSFI domains using the same tests mentioned above. A complementary analysis was undertaken dichotomizing samples using the cutoff 26.0 in the FSFI general score to evaluate the sexual dysfunction percentage. The non-parametric tests used were the Yates chi-square test (independent samples) and the Cochran and McNemar tests (related samples). Differences were considered statistically significant when p<0.05. The total FSFI scores during the first 22.60.4, as compared with, 23.80.7 during the second, and 17.50.2 during the third trimesters of pregnancy.

There was no difference in the sexual function results in the first and second trimesters, but there was a significant difference in the median scores in the FSFI domains throughout pregnancy, specifically in the third trimester. In this case, there was a significant decrease in the score of all FSFI domains when compared to the second trimester (p<0.01). We investigated the prevalence of sexual dysfunction in the pregnant women, based on the total FSFI scores, using the recommended clinical cutoff of 21 (19). More than 60% of pregnant women were categorized as potentially having sexual dysfunction using the lower threshold of 21. The prevalence of female sexual dysfunction in the first trimester was (64.22), in the Second trimester (70.73) and in the third trimesters (87.8%) (Table 1).

**Table 1:** Prevalence of female sexual dysfunction stratified by trimesters (N = 123)

Trimesters Prevalence	N (%)	95% Confidence interval
**First**	79(64.22)	55.0% to 62.1%
**Second**	87(70.73)	58.0% to 64.3%
**Third**	108(87.8)	78.0% to 82.9%

When the domain scores were compared according to each trimester of pregnancy, There was a significant decrease in the score of all FSFI domains in the third trimester when compared to the first trimester (p<0.05). Both, the full-scale score, and the scores on all 6 of the individual domains, differed significantly among women in each of the 3 trimesters of pregnancy. The FSFI scores changed in accordance with gestational age. During the second trimester, the FSFI scores in domain: desire, arousal and lubrication increased, while in domain orgasm, Satisfaction and pain decreased but without statistical significance. The mean full-scale score declined progressively from the first trimester to the third trimesters of pregnancy (Table 2).

**Table 2:** FSFI Domain scores during the trimesters of pregnancy (mean SD)

FSFI domain	Trimester 1	Trimester 2	Trimester 3
**Desire**	3.41.1	3.60.6	2.70.3*
**Arousal**	4.60.1	4.80.9	3.10.9*
**Lubrication**	4.70.7	5.10.9	3.70.6*
**Orgasm**	4.10.3	3.61	2.71.6*
**Satisfaction**	3.70.6	3.10.9	2.91.4
**Pain**	4.10.1	3.61.4	3.20.7
**Total Score**	22.60.4	23.80.7	17.50.2

* *P*<0.05 for all domains in the Friedman test

In contrast, no significant difference was found between full-scale scores of participants in the second and third trimesters (p=0.247). Meanwhile, during the third trimester the scores of every domain, especially in domain desire, arousal, lubrication and orgasm significantly decreased (p <0.05) (Table 3).

**Table 3:** IIEF Domain scores during the trimesters of pregnancy (mean s.d.)

IIEF domain	Trimester 1	Trimester 2	Trimester 3
**Erectile**	25.471.4	25.60.3	25.70.5
**Orgasm**	8.40.2	8.40.7	7.80.6
**Desire**	8.850.7	8.00.3	7.10.2*
**Overall satisfaction**	9.120.5	9.60.3	8.21.6*
**Sexual Satisfaction**	13.20.6	13.70.7	11.90.2*

* *P*<0.05 for all domains in the Friedman test

Correlation evaluated, showing that the overall FSFI and IIEF scores were forcefully correlated (r = 0.51, p < 0.02) IIEF scores were significantly correlated with scores on all domains of FSFI (all p < 0.04). The correlation between Female sexual arousal and sexual satisfaction domains with total score IIEF was highest (r = 0.42 and r = 0.37). The correlation among the scores of all domains of IIEF, male intercourse satisfaction domain had largest the correlation (r = 0.51) with the FSFI total score (Table 4).

**Table 4:** Pearson correlation coefficients between IIEF and domains of FSFI

	Male erectile	Male orgasm	Male desire	Sexual Satisfaction	Overall satisfaction
**Female sexual** **desire**	0.15	0.21	0.24	0.21	0.22
**Female sexual** **arousal**	0.42	0.42	0.38	0.37	0.41
**Female vaginal** **lubrication**	0.49	0.52	0.39	0.44	0.53
**Female orgasm**	0.53	0.51	0.33	0.48	0.46
**Female sexual** **satisfaction**	0.47	0.39	0.35	0.48	0.53
**Female sexual pain.**	0.62	0.49	0.36	0.69	0.51

## Discussion

It is widely accepted that pregnancy has some influences on the sexual behavior of women^26^. A decline in sexual activity is reported during pregnancy^9^,^11^,^18^. The physiologic and psychological changes that occur during pregnancy may affect sexual function and sexual activity^10^. Sexual function among pregnant women have been extensively studied in the literature^1^,^4^,^9^. The FSFI was chosen for this study because it is a specific and multidimensional research instrument, following the evolution of new concepts of female sexual dysfunction^16^. It also allowed comparison of our results with other studies using similar methodology for pregnant samples. Results of several studies about the association between pregnancy and sexual dysfunction could not be directly compared because different definitions and methods were used to evaluate the sexual function during gestation. However, prospective studies assessing the sexual function among couples during pregnancy are limited^27^. In our study, we have designed a prospective cohort study to evaluate sexual function among couples during pregnancy and we have found significant changes in male and female sexual functions. In 2011, a prospective study published by Ahmadi et al^28^ evaluated the sexual health of 40 women during their pregnancies and reported significant decreases in all domains of the FSFI during pregnancy, especially during the later phases of the pregnancy. In 2014, Sossa et al^29^ performed a descriptive correlational study to evaluate the changes in sexual function throughout pregnancy. Their findings were consistent with those of similar studies, showing a significant reduction in FSFI scores from the first to the third trimesters. The pregnant women showed a significant decrease in all domains of the FSFI, including full-scale scores between the first and third trimesters. The final trimester of pregnancy is characterized by significant changes in the woman's body. These changes could be the reason for the decrease in libido and sexual activity during this period^30^.

An increase of both the abdominal volume and fetal weight cause lack of balance and compensatory postural changes, forcing the female organism to begin using muscles seldom used before pregnancy, which can cause lumbar pain a specific symptom of the gestational period's end^23^. Also, fatigue, anxiety and the natural fear felt due to proximity of labor tend to make the sexual relationship unattractive for pregnant women. Another factor that contributes to decrease the female sexual function is the partner's loss of sexual interest because of worries with the woman and the baby, as well as the non-erotic effect of the woman's appearance at the end of pregnancy. In the present work, changes in the scores of all domains were not significant between the first and the second trimesters. These results were also observed by Sossa et al, who evaluated pregnant women sexual function using the FSFI throughout pregnancy^29^.

The FSFI scores in domain: desire, arousal and lubrication increased from the second trimester to the first trimesters. The second gestational trimester is considered the most emotionally stable periods of gestation when pregnancy seems to be clearly established diminishing, this way, fear of fetal loss (4, 9). Reaffirmation of femininity through, the duo woman/maternity associated with the pregnant pelvis vascular changes and with the cessation of nausea allows an increase in orgasmic quality as well as in the level of erotism. These factors can explain the presence of the sexual function's best indicators in the second trimester, which was already mentioned in this article.

A diagnosis of sexual dysfunction could be made in more than 60% of participants using a cutoff score of 21^31^, and could be made in more than 90% if a cutoff of 26.55^24^ is used. This may indicate either a truly high prevalence of sexual dysfunction among Iranian. This may indicate either a truly high prevalence of sexual dysfunction among Iranian women. These averages for pregnant women were below the suggested cutoffs for diagnosis of sexual dysfunction (<26.55),^24^ no other studies had determined an appropriate cutoff score to diagnose sexual dysfunction using the FSFI in Iranian pregnant women and Iranian non-pregnant women (<21). Using IIEF and FSFI, our prospective study is the first to show the influence of pregnancy on couple's sexual function. A number of reports exist in the literature to correlate with our findings^6^, ^13^,^28^. Sexual interest in pregnant women was decreased in the first trimester, Increased in the second trimester and decreased at the end of the third trimester but in male was reported to be variable or decreased.

## Conclusion

We expected results of this study to caution health providers, to pay attention to sexual problems of pregnant women. They must take steps to prevent or treat the sexual problems of pregnant women. It is unacceptable that health specialists neglect to complain about this issue.

## Limitations

Although we believe this study contributes valuable information to the study of sexual function in couples, it also presents several limitations. The participants consisted solely of women who attended out-patient clinics of Public Health Centers. Thus, our sample may not be representative of the Iran population, although the attendees of these teaching centers represented different social classes. In addition, the Rasht, where this Health Centers were situated, comprises a mixed population from various parts of Iran. Nevertheless, a community-based sampling approach may result in a more representative sample. However, given the sensitive nature of this topic, such a sampling method may not be feasible. Further, Iranian women are not inclined to talk about their sexual habits or behavior, and many taboos persist in this population. Therefore, we were forced to restrict our assessment of the sexual activities of women, to those who participated voluntarily in this study.

## Recommendation

It is recommended that future studies should avoid these deficiencies, in order to enhance the understanding of the issue of sexual dysfunction in pregnant women and achieve an accurate assessment of this problem in the Iran population.
